# Evaluation of total oxidant and antioxidant status in dogs under different CO_2_ pneumoperitoneum conditions

**DOI:** 10.1186/s13028-015-0113-3

**Published:** 2015-05-08

**Authors:** Jae Yeon Lee, Seok Hwa Choi

**Affiliations:** Veterinary Medical Center, Chungbuk National University, Cheogju, 361-763 Korea

**Keywords:** Co_2_ pneumoperitoneum, Insufflation pressure, Laparoscopic surgery, Oxidative stress, Dogs

## Abstract

**Background:**

The induction of the pneumoperitoneum increases intraabdominal pressure (IAP), causing splanchnic ischemia, whereas its deflation normalizes IAP and splanchnic blood flow. We investigated the oxidant-antioxidant status of dogs who underwent low pressure (7 mm Hg), standard pressure (12 mm Hg), and high pressure (15 mm Hg) pneumoperitoneum.

**Results:**

Twenty-four beagle dogs (12 males and 12 females), 4–6 years old, weighing 8–11 kg were used. The animals were assigned to one of four groups (n = 6 dogs). Group 1 served as a control; these animals received only anaesthesia for 90 min. In groups 2, 3 and 4, intra-abdominal pressure was increased to 7, 12 and 15 mmHg, respectively, and maintained for 60 min. Total oxidant status (TOS) and total antioxidant status (TAS) were determined in venous blood samples. The percentage ratio of TOS to TAS provided an oxidative stress index (OSI).

No significant difference in TOS levels was found among the groups. A significant decrease in TAS levels and an increase in OSI levels were observed at 90 min and 24 h of pneumoperitoneum deflation within group 4. No differences were found among the groups.

**Conclusions:**

A high pressure pneumoperitoneum induced significant changes in TAS and OSI. In addition, TOS and TAS levels are useful markers for evaluating changes in the oxidative status caused by a pneumoperitoneum during laparoscopy. Furthermore, a low-pressure pneumoperitoneum could attenuate oxidative stress induced by CO_2_ insufflation in dogs.

## Background

A pneumoperitoneum is generally established during a laparoscopy by continuous insufflation of carbon dioxide (CO_2_) into the peritoneal cavity to provide adequate visualization and exposure of structures. Laparoscopic surgery has many advantages for the patient compared to conventional surgery, including smaller incisions, less postoperative pain, faster discharge, and a better cosmetic effect [1,2]. However, despite its benefit, a CO_2_ pneumoperitoneum is not free of adverse events. Oxidative stress is the general phenomenon of oxidant exposure and antioxidant depletion, or oxidant-antioxidant imbalance. Over the last several decades, it has become evident that oxidative stress, usually in the form of reactive oxygen species (ROS), is produced during metabolic and physiological processes [3,4]. Oxidative stress can be quantified by measuring the levels of total antioxidant capacity. Total oxidant status (TOS) and total antioxidant status (TAS) are often used to estimate the overall antioxidant status [5,6].

Experimental and clinical studies have shown that an increase in intra-abdominal pressure (IAP) associated with a pneumoperitoneum greater than normal physiological portal pressure (7–10 mm Hg) causes splanchnic ischemia [7], which leads to free radical production [8]. Although abdominal deflation at the end of laparoscopic procedures reduces IAP and increases splanchnic perfusion, oxidative stress from the ischemic injury remains [9]. Therefore, to minimize oxidative stress during a pneumoperitoneum, an IAP less than the portal pressure may be a useful method to reduce ischemic injuries. Various animal and human studies have investigated the ischemia-reperfusion injury that occurs as a result of a pneumoperitoneum [8,10-12], but no controlled studies have been conducted to evaluate oxidant-antioxidant status during a pneumoperitoneum in dogs.

Our purpose was to evaluate the oxidant-antioxidant status of dogs insufflated with CO_2_ at low pressure (7 mm Hg), standard pressure (12 mm Hg), and high pressure (15 mm Hg). Our hypotheses were (1) a CO_2_ pneumoperitoneum induce the oxidative stress in dogs; (2) the low pressure pneumoperitoneum in dogs gives a beneficial effect on the oxidant-antioxidant status.

## Methods

### Animals and anesthesia

Twenty-four beagle dogs (12 males and 12 females), 4–6 years old, weighing 8–11 kg were used. All dogs were healthy based on clinical examinations. The experimental and housing protocols were approved by the Chungnam National University Animal Care and Use Committee (approval no. 2010-2-37). This study was conducted 14 days after procuring the dogs. The dogs were kept in a quiet room to avoid any stress-inducing factors during this period. Food was withheld for 8–12 h prior to anesthesia, but access to water was allowed.

The dogs were premedicated with atropine sulfate (0.04 mg/kg, SC), and an analgesic agent (butorphanol, 0.2 mg/kg, IM) before the induction of anesthesia. Anesthesia was induced with propofol (6 mg/kg) and 100% oxygen. Animals received 6 mg/kg of IV propofol under pure oxygen. After endotracheal intubation, anesthesia was maintained with isoflurane, at an end-tidal concentration of 2%, using a Datex ohmeda ADU anesthesia system (semi-closed system, Helsinki, Finland), with an oxygen flow rate of 2 L/min. After anesthesia was stabilized, the animals were placed in a supine position, and the abdominal skin was shaved and cleansed with povidone-iodine solution. A pneumoperitoneum was created in all animals, except the control group, by inserting a standard Veress needle at 1 cm caudal to the umbilicus on the middle line. Placement of the needle tip into the peritoneal cavity was confirmed by the saline drop test. CO_2_ was insufflated using an automatic device (UHI®, Olympus, USA) at a rate of 1–2 L/minute until the IAP reached 7, 12, or 15 mm Hg (±1 mm Hg), which was maintained for 60 minutes. After a 15miniute stabilization period all baseline measurements were performed. During anesthesia, heart rate (HR), mean arterial pressure (MAP), rectal temperature (RT), respiratory rate (RR), saturation of peripheral oxygen (SpO_2_) and end-tidal CO_2_ (ETCO_2_) were measured using a Datex-Ohmeda Anesthesia Monitor system (Helsinki, Finland), and the dogs were given IV fluid (Hartmann’s solution, 10 ml/kg/h).

### Study groups

The animals were assigned to one of four groups (n = 6 dogs). Group 1 served as the control; these animals received only anesthesia for 90 min. In groups 2, 3, and 4, IAP was increased to 7, 12, and 15 mmHg, respectively, and maintained for 60 min. Total oxidant status (TOS) and total antioxidant status (TAS) were determined in venous blood samples before induction of anesthesia (T0), at 30 minutes after peritoneum setting (T1), at the end of the peritoneum setting and before deflation (T2), 30 minutes after deflation (T3), and 24 hr after deflation (T4). In group 1, the same parameters were determined before anesthesia, at 30, 60, 90 min of anesthesia and at 24 hr after anesthesia recovery.

### Measuring TOS, TAS and calculating OSI

Approximately 3 ml blood was collected into a plasma separation tube containing heparin for analysis of oxidative stress markers. Blood samples were centrifuged at 3000 rpm for 10 min to separate plasma, and the plasma samples were stored at −80°C until analysis. Plasma TOS and TAS levels were determined using a commercially available kit developed by Erel [5,6]. The results of TOS and TAS are expressed in micromolar hydrogen peroxide equivalents per liter (μmol H_2_O_2_ equiv/L) and mmol Trolox equiv/L, respectively. The ratio of TOS to TAS provided the oxidative stress index (OSI), an indicator of the degree of oxidative stress.

### Statistical analysis

Values are expressed as means and standard deviation. Kruskal-Wallis analysis of variance was used for group comparison. Between-group differences were compared by Mann–Whitney *U*-test. The Friedman and Wilcoxon tests were used for within-group data analysis. The level of statistical significance was set at p-value < 0.05. All statistics were performed with SPSS ver. 18.0 (SPSS, Chicago, IL, USA).

## Results

**Figure 1:**
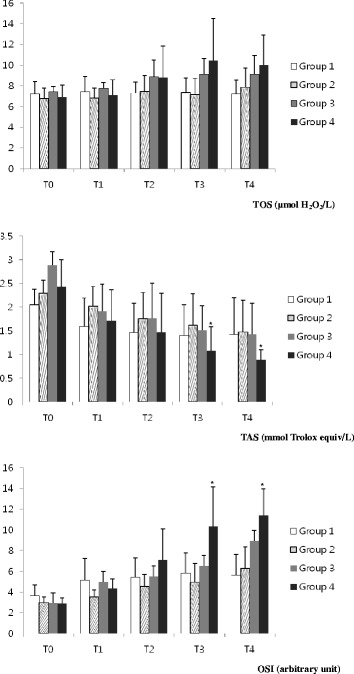
Plasma levels of oxidative stress parameters in dogs. The values represent the mean ± SD (n = 6). *Statistically difference compared to baseline values.TOS: total oxidant status, TAS: total antioxidant status, OSI: oxidative stress index, T0: before induction of anesthesia; T1: at 30 minutes after pneumoperitoneum; T2: at the end of the pneumoperitoneum and ischemia but before deflation; T3: 30 minutes after deflation; T4: 24 hours after deflaztion.

All dogs were hemodynamically stable throughout the procedure and completed the study. There were no significant worsening in heart rate, mean arterial pressure, rectal temperature, end-tidal carbon dioxide, peripheral oxygen saturation, and respiratory rates (Table 1). The plasma TOS, TAS, and OSI are shown in Figure 1. Increases in plasma TOS and OSI levels, and a decrease in TAS levels were observed in group 2, 3, and 4 after creating the pneumoperitoneum. No significant difference in TOS levels was observed within groups or among the groups, but significant changes in the plasma TAS and OSI levels were observed within group 4. In group 4, the TAS levels at T3 and T4 were significantly decreased from T0 (p = 0.029). In group 4, the OSI levels at T3 and T4 were significantly increased from T0 (p = 0.029). No differences in TOS, TAS and OSI levels were found when the groups were compared.

**Table 1:** **Heart rate (HR), mean arterial pressure (MAP), rectal temperature (RT), respiratory rate (RR), saturation of peripheral oxygen (SpO**_**2**_ **) and end-tidal CO**_**2**_ **(ETCO**_**2**_ **) in dogs**

**Parameter**	**Group**	**Pre (t0)**	**15 min**	**30 min (t1)**	**45 min**	**60 min (t2)**	**90 min (t3)**
HR (breath/min)	Group 1	110.0 ± 14.3	113.4 ± 16.9	115.6 ± 15.3	114.4 ± 15.9	111.5 ± 16.3	115.4 ± 20.9
	Group 2	117.5 ± 11.9	119.5 ± 15.7	119.5 ± 17.6	123.8 ± 10.0	128.8 ± 14.1	125.9 ± 16.0
	Group 3	120.5 ± 10.5	121.3 ± 17.2	122.3 ± 19.1	124.2 ± 19.5	120.3 ± 15.5	127.6 ± 17.3
	Group 4	117.7 ± 16.1	120.5 ± 10.8	122.3 ± 10.2	121.2 ± 18.3	120.5 ± 15.3	125.2 ± 11.3
MAP (mmHg)	Group 1	85.8 ± 9.5	88.5 ± 5.9	90.0 ± 10.0	91.8 ± 12.9	88.9 ± 8.9	90.5 ± 15.0
	Group 2	87.7 ± 6.5	86. 5 ± 9.9	86.0 ± 11.0	87.8 ± 17.0	87. 5 ± 6.5	88. 5 ± 9.3
	Group 3	85.8 ± 7.5	88.5 ± 5.9	85.0 ± 15.0	86.4 ± 15.1	87.5 ± 5.6	88.5 ± 15.9
	Group 4	83.4 ± 10.5	84.5 ± 11.9	82.0 ± 9.0	80.5 ± 11.9	84.3 ± 15.9	85.5 ± 16.9
RT (°C)	Group 1	38.7 ± 1.4	38.6 ± 1.4	38.6 ± 1.4	38.5 ± 1.4	37.6 ± 0.4	37.5 ± 1.4
	Group 2	38.5 ± 0.4	38.4 ± 0.8	38.4 ± 0.8	38.4 ± 0.8	37.3 ± 1.8	37.4 ± 1.2
	Group 3	37.7 ± 1.1	37.6 ± 1.2	37.5 ± 1.5	37.5 ± 1.5	37.2 ± 1.8	37.0 ± 1.0
	Group 4	37.5 ± 1.6	37.5 ± 1.4	37.4 ± 1.6	37.4 ± 0.9	37.0 ± 1.9	37.2 ± 1.5
RR (breath/min)	Group 1	10.0 ± 4.34	13.4 ± 6.99	15.6 ± 5.32	14.4 ± 5.94	16.4 ± 5.94	13.5 ± 6.12
	Group 2	17.5 ± 11.98	19.5 ± 5.72	19.5 ± 7.69	23.8 ± 10.05	20.8 ± 15.11	25.6 ± 11.04
	Group 3	20.5 ± 10.56	21.3 ± 7.23	22.3 ± 9.18	24.2 ± 9.54	25.2 ± 5.54	26.3 ± 7.34
	Group 4	17.7 ± 6.19	20.5 ± 10.80	22.3 ± 10.25	21.2 ± 8.38	25.4 ± 7.68	26.2 ± 9.14
ETCO_2_ (mmHg)	Group 1	42.3 ± 5.14	48.0 ± 5.37	44.8 ± 5.34	45.8 ± 4.88	44.7 ± 5.86	45.7 ± 3.21
	Group 2	41.5 ± 2.73	51.2 ± 8.13	51.5 ± 9.03	46.5 ± 4.64	44.6 ± 5.74	45.5 ± 6.34
	Group 3	41.3 ± 12.40	47.5 ± 7.23	52.0 ± 9.40	51.3 ± 8.14	51.3 ± 8.14	53.3 ± 5.14
	Group 4	42.1 ± 13.90	55.5 ± 8.87	56.8 ± 6.89	53.8 ± 6.28	50.8 ± 9.28	50.5 ± 7.28
SpO_2_ (%)	Group 1	100 ± 0	100 ± 0	100 ± 0	100 ± 0	100 ± 0	100 ± 0
	Group 2	100 ± 0	100 ± 0	100 ± 0	100 ± 0	100 ± 0	100 ± 0
	Group 3	100 ± 0	100 ± 0	100 ± 0	100 ± 0	100 ± 0	100 ± 0
	Group 4	100 ± 0	100 ± 0	100 ± 0	100 ± 0	100 ± 0	100 ± 0

The values represent the mean ± SD (n = 6).

Variables were measured before induction of anesthesia (pre) and at 15, 30, 45, 60, and 90 minutes after induction of anesthesia. T0: before induction of anesthesia; T1: at 30 minutes after pneumoperitoneum; T2: at the end of the pneumoperitoneum and ischemia but before deflation; T3: 30 minutes after deflation.

## Discussion

Laparoscopic surgeries are increasingly performed in veterinary clinics. However, the possible unwanted side effects of these procedures remain unclear. This study showed that at an IAP of 15 mm Hg there were significant changes in TAS and OSI whereas no significant changes were observed in theses parameters at 0, 7 or 12 mm Hg.

Previous studies in humans have shown that a pneumoperitoneum during laparoscopic surgery causes a significant reduction in gut perfusion and microcirculation [13,14]. Deflation of the pneumoperitoneum reduces the IAP and increases splanchnic perfusion. Thus, a pneumoperitoneum for laparoscopic surgery may be a representative ischemia-reperfusion model. ROS generation is the main cause of ischemia-reperfusion injury in various tissues [14], and ROS have been implicated strongly in the pathogenesis of cellular damage associated with ischemia-reperfusion injury [15].

Evaluating oxidative stress can indirectly reflect changes in microcirculation in the splanchnic area during laparoscopic insufflation. Many oxidative stress markers, including nitric oxide, lipid peroxidation, protein carbonyl content, and protein sulfhydryl have been investigated [15]. In addition to these stress parameters, plasma TOS and TAS levels have been used to reflect oxidative stress and actions against oxidative stress, respectively [5,6].

Hydrogen peroxide and other peroxide derivatives produced at physiological concentrations and at higher concentrations under some pathological conditions, diffuse into plasma [5]. The level of total peroxide was measured and expressed as TOS in this study. Evaluating oxidative stress can indirectly reflect changes in organ microcirculation during surgery. The major findings of the present study were that OSI was higher after induction of the pneumoperitoneum compared to those at baseline. TOS and OSI levels tended to be higher and TAS levels tended to be lower under high pressure (15 mmHg) insufflation compared with those in the other groups, but the differences were not significant.

Many antioxidant molecules in blood plasma inhibit the oxidant reaction that produce free radical. Therefore, these antioxidants in plasma can protect the organs against oxidative stress [16]. In the present study, a significant decrease in TAS levels was observed at T3 and T4 in the plasma under the high pressure pneumoperitoneum conditions.

Plasma TAS levels were measured using an automated colorimetric measurement method which is based on the bleaching of color characteristics of a more stable ABTS (2, 2′-azino-bis [3-ethylbenzothiazoline-6-sulfonic acid]) radical cation by antioxidants (Erel [6]). In a previous report by Glantzounis et al. [17], plasma TAS levels decreased significantly postoperatively compared to those preoperatively during laparoscopic surgery. Our study showed that the decreased plasma TAS levels when pneumoperitoneum setting was performed in dogs at 15 mm Hg rather than 12 mm Hg or less, although this was not statistically significant between groups. The findings in this study and a previous report suggest that a lower plasma TAS level may reflect changes in the antioxidant status caused by a pneumoperitoneum during anaesthesia [17]. These results indicate the consumption of plasma antioxidants by ROS generated during the operation.

Plasma TOS levels were also determined by measuring hydrogen peroxide, which occurs in higher concentrations under some pathogenic conditions [8]. In the present study, a significant increase in TOS levels was not found after a pneumoperitoneum, but levels of TOS rose in a time-dependent manner with increased IAP. Other study has shown similar patterns of TOS levels in human laparoscopic surgery [12,18].

The OSI was higher after the pneumoperitoneum than that before the pneumoperitoneum, and a significant increase in OSI levels was observed at T3 and T4 in group 4. These significant changes in OSI levels may be associated with significant changes in plasma TAS levels.

Splanchnic vasoconstriction is due to several mechanisms. Vasopressin, produced by a central nervous system pathway in response to increased IAP, constricts renal, superior mesenteric and celiac vasculature [19-22]. Insufflation pressure is generally set at 12–15 mmHg, which raises IAP pressure above that of normal portal circulation in humans [23]. The mesenteric vasoconstriction causes a significant reduction in organ perfusion and reduced portal venous flow during routine laparoscopic procedures [8,13,24]. Given the increasing complexity and length of laparoscopic procedures, organ ischemia-reperfusion injury and oxidative stress associated with a pneumoperitoneum may become a more severe problem. In the present study, oxidative stress was multifactorial in origin; the main impacts were from ischemia-reperfusion events due to the pneumoperitoneum and anesthesia. The change in oxidative parameters in group 1 was thought to be due to the anesthesia itself. However, there are a few limitations to this study that deserve consideration. Owing to the various interactions among antioxidants and oxidants, the lack of test sensitivity, effects of anesthesia, and the ability of the body to maintain a redox potential we cannot be certain that OSI reflects the redox balance between oxidation and antioxidation by pneumoperitoneum in this study.

Various animal and human studies have investigated the ischemia-reperfusion injury that occurs as a result of a pneumoperitoneum [7,8,10-12]. There is clear evidence from animal studies that a pneumoperitoneum results directly in end-organ ischemia and injury. A recent study by Nickkholgh [25] demonstrated that a 12 mmHg pneumoperitoneum for 90 min induced histological and biochemical evidence of organ reperfusion injury in rats. Other study has shown similar patterns of biochemical changes following a pneumoperitoneum [26]. Our results showed a trend towards increased oxidative stress markers in dogs at an insufflation pressure of 15 mmHg compared to that in the other groups, but the difference was not statistically significant. Although increased plasma TOS and OSI levels were observed in this study, the changes in oxidative parameters in the pneumoperitoneum groups were not significantly different from those in the control group, and the cardiopulmonary parameters were stable during the pneumoperitoneum in all groups. Therefore, insufflation pressures ranging from 7– 15 mmHg can be applied in dogs for the laparoscopic surgery. However, the group of animals treated with low-pressure pneumoperitoneums showed less oxidative stress compared with the high-pressure pneumoperitoneum group. Significant changes of TAS and OSI levels were observed within group 4. Therefore, a low pressure CO_2_ pneumoperitoneum should be considered for patients with compromised splanchnic function, particularly those undergoing prolonged laparoscopic surgery. In addition, use of exogenous antioxidants including vitamins C, and E (alpha-tocopherol) can be the potential means to minimize free radical-induced complication during laparoscopic surgery.

## Conclusions

A CO_2_ pneumoperitoneum induced changes in total oxidant and antioxidant status of dogs in this study. In addition, the results showed that low insufflation pressure during a CO_2_ pneumoperitoneum tended to produce less oxidative stress compared with standard or high insufflaton pressure, although these differences did not reach statistical significance. Further investigation is needed to evaluate the clinical outcome in patients with a CO_2_ pneumoperitoneum undergoing laparoscopic surgery.
